# Effect of Betaine on Reducing Body Fat—A Systematic Review and Meta-Analysis of Randomized Controlled Trials

**DOI:** 10.3390/nu11102480

**Published:** 2019-10-16

**Authors:** Xiang Gao, Huijun Zhang, Xiao-fei Guo, Kelei Li, Shan Li, Duo Li

**Affiliations:** 1 Institute of Nutrition and Health, College of Life Sciences, Qingdao University, Qingdao 266071, China; gaoxiang@qdu.edu.cn (X.G.); 2018020870@qdu.edu.cn (H.Z.); jackic1103@163.com (X.-f.G.); likelei@qdu.edu.cn (K.L.); lishanlhw@163.com (S.L.); 2 Department of Food Science and Nutrition, Zhejiang University, Hangzhou 310058, China

**Keywords:** betaine, body fat, obesity, clinical trial, meta-analysis

## Abstract

Animal studies have shown the beneficial effect of betaine supplementation on reducing body fat, while the data from human studies are controversial and inconsistent. The objective of the present systematic review was to investigate the effects of betaine intervention on treating obesity in humans and quantitatively evaluate the pooled effects based on randomized controlled trials with a meta-analysis. The PubMed and Scopus databases, and the Cochrane Library, were searched up to September 2019. Weighted mean differences were calculated for net changes in obesity-related indices by using a random-effects model. Publication bias was estimated using Begg’s test. Six studies with 195 participants were identified. Betaine supplementation significantly reduced the total body fat mass (−2.53 kg; 95% CI: −3.93, −0.54 kg; I^2^ = 6.6%, P = 0.36) and body fat percentage (−2.44%; 95% CI: −4.20, −0.68%; I^2^ = 0.0%, P = 0.44). No changes were observed regarding body weight (−0.29 kg; 95% CI: −1.48, 0.89 kg; I^2^ = 0.00%, P = 0.99) and body mass index (−0.10 kg/m^2^; 95% CI: −5.13, 0.31 kg/m^2^; I^2^ = 0.00%, P = 0.84). The results suggested that dietary betaine supplementation might be an effective approach for reducing body fat.

## 1. Introduction

Excess body fat accumulating in the body leads to obesity, which is recognized as a chronic disease and is correlated with multiple health problems, such as type 2 diabetes, cardiovascular diseases, and cancers [1,2]. Over the past decades, the prevalence of overweight and obesity has increased substantially and now affects over 600 million people worldwide [3]. Currently, effective weight reduction and maintenance strategies, such as lifestyle modification or dietary intervention have been paid increasing attention. Of these, supplementary dietary nutrients from natural products have achieved a great performance in the prevention of obesity.

Betaine is a zwitterionic quaternary ammonium compound, a methyl derivative of glycine and a byproduct isolated from molasses during sugar beets refinement. In addition to sugar beets, betaine is abundant in many other foods, such as spinach, whole grains and seafood. In adults, daily average betaine intake is approximately 100–400 mg [4]. When healthy subjects orally take betaine, it is freely filtered in the kidney, re-absorbed into the blood circulation, and either absorbed and stored in tissues as an organic osmolyte to protect cells under stress or as a catabolic source of methyl groups via transmethylation of homocysteine (Hcy) [5]. In addition to direct dietary intake of betaine or betaine-containing foods, humans can also obtain betaine via choline oxidation in the liver and kidney [6]. To date, numerous studies have discussed the relationships between betaine treatment and human health. Betaine supplementation was reported to be beneficial to human health, including preventing cancers [7,8], improving liver function [9], decreasing serum homocysteine levels [10] and attenuating chronic inflammation [5]. 

The favorable effect of betaine on reducing body fat has been proved in animals, such as rodents [11], pigs [12] and fowls [13]. In livestock husbandry, betaine has also been used as a feed additive for reducing carcass body fat percentage [14]. However, data from humans are rare. Few randomized controlled trials (RCTs) have been conducted to evaluate supplemental betaine on obesity, while the results are inconsistent. In a study examining 42 obese subjects with 6 g/day betaine treatment for 12 weeks (2002) [15] and another study involving 17 young males with 2 g/day betaine supplementation for 10 days (2012) [16], no change of obesity-related indices were found. Meanwhile, 4 g/day betaine treatment for 24 weeks also had no effect on body weight and BMI in healthy young subjects [17]. In 2013, a study by Cholewa et al. first proved in 11 young and lean males that a 2.5 g/day betaine supplementation with a 6-week progressive resistance training program significantly improved body composition (reduced fat mass and increased lean mass) [18]. A systematically review was subsequently published by the same group in 2014 and concluded that there are potential beneficial effects of betaine on body composition in humans [19]. Recently, two RCTs, investigating the effect of betaine supplementation on reducing body fat, were published in 2018. Cholewa et al. showed 2.5 g supplementary betaine per day with 9 weeks exercise training in 11 young women decreased body fat more than 12 placebo treatment subjects [20]. Grizales et al. found 3.3 g betaine twice orally for 10 days, then 4.95 g betaine twice orally for 10 days in 14 prediabetes and obese participants showed a reduction in body weight and BMI compared with 13 participants treated with placebo, even though the trend was not significant [21]. The results of these reports regarding the effects of betaine on obesity or improving body composition in humans need to be objectively reviewed and summarized. 

Therefore, to provide an up-to-date evaluation of the roles of betaine in obesity, we systematically reviewed the literature and conducted a meta-analysis on RCT studies regarding betaine and obesity or body composition in humans. We hypothesized that betaine treatment reduces body fat in humans.

## 2. Materials and Methods 

The present study was performed according to the recommendations of the Preferred Reporting Items for Systematic Reviews and Meta-Analysis (PRISMA) statement [22].

### 2.1. Literature Search

A systematic literature search was performed up to Sep 2019 with the PubMed (http://www.ncbi.nlm.nih.gov/pubmed), Scopus (http://www.scopus.com) and Cochrane Library (http://www.thecochranelibrary.com) databases. Betaine was used as a search term paired with the following words: obesity, body composition, metabolic syndrome, body weight, body mass index (BMI), waist circumference, body fat and body fat percentage. All searches were restricted to English-language publications. Meanwhile, a manual search was also conducted by scrutinizing the reference lists of original articles, recent reviews and meta-analyses by using Google and Baidu Scholar.

### 2.2. Inclusive Criteria 

A trial that met the following criteria was incorporated in the present study: (1) conducted in adults; (2) RCTs of either parallel or cross-over design; (3) used betaine as the only intervention in subjects; and (4) provided the available data to calculate the mean differences between baseline and endpoint for obesity-related indices, including body weight, BMI, waist circumference, body fat, and body fat percentage.

### 2.3. Data Extraction and Quality Assessment

Data extraction was independently conducted by two investigators (X.G. and H.Z.), and any discrepancy was resolved via discussion. The basic information of the eligible trials was extracted, such as surname of first author, published year, region/nation, mean age of participants, sample-size, gender, types of intervention and duration of intervention. For each trial, the means and standard deviations (SDs) of obesity-related indices at baseline and endpoint in both the control and intervention groups were extracted. For studies with multiple time points for the same participants, only the last endpoint was used for analysis. If the trial did not provide the SDs directly, we calculated them from standard error of the mean (SEM) or 95% confidence interval (CI) by using the equation listed in the Cochrane handbook [23]. 

Quality assessment for RCT trials was carried out with Jadad score criteria [24], including the following items: (1) randomization; (2) random sequence generation; (3) reporting the reasons for withdraws and dropouts; (4) blinding and (5) allocation concealment. The trials scored one point for each aspect reported. Trials with Jadad score ≥ 4 were classified as high quality.

### 2.4. Data Analysis

A random-effects model described by DerSimonian and Laird was performed to calculate the pooled effects of and corresponding 95% CI for each obesity-related index [25]. Heterogeneity among studies was assessed with I^2^. The I^2^ represented the proportion of total variation, and the low, moderate and high degrees of heterogeneity were defined based on values of 25%, 50% and 75% as cut-off points [26]. We determined that there was no significant heterogeneity among trials when I^2^ < 50%. Begg’s test was adopted to examine publication bias with a significant level at P < 0.1 [27]. Statistical analysis was conducted using STATA 11.0 for windows (Stata CORP, College station, TX, USA). Two-tailed *p*-value < 0.05 was considered as significant.

## 3. Results

### 3.1. The Process of Study Selection

**Figure 1:**
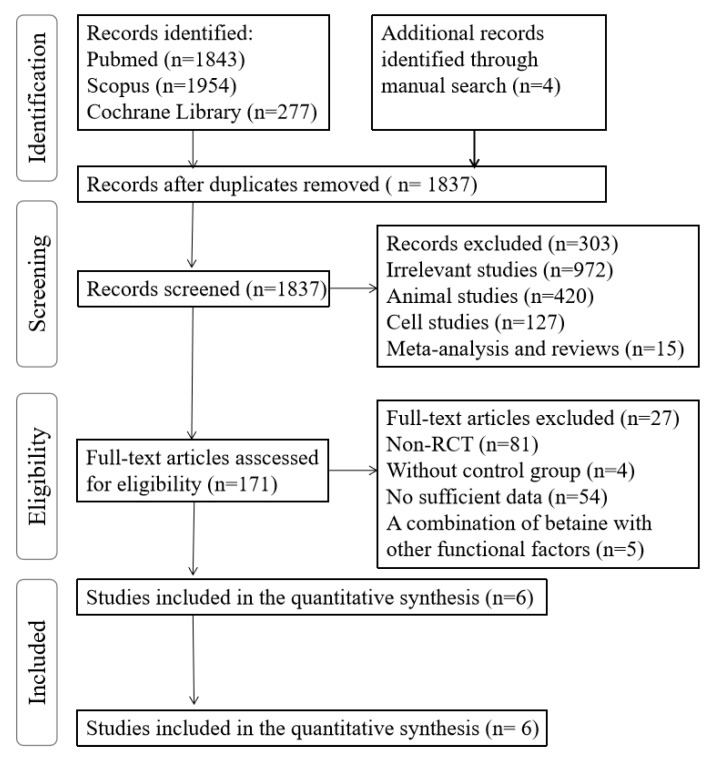
Flowchart of the literature search.

The literature search result is presented in Figure 1. A total of 1837 potentially relevant titles and abstracts were identified from PubMed, Scopus and the Cochrane Library. After ruling out animal studies, cell studies and those irrelevant to the aim of this meta-analysis, six articles were found to be eligible for data synthesis [15,16,17,18,20,21].

### 3.2. The Characteristics of Inclusive Studies

The basic information of the included studies is listed in Table 1. Three trials were conducted in USA [18,20,21], and three other trials were carried out in Finland [15,17], and Brazil [16], respectively. Two trials used training programs during the betaine supplementation period [18,20] and four others were instructed not to exercise [15,16,17,21]. On the basis of Jadad scoring criteria, five trials were regarded as high-quality.

### 3.3. Betaine Supplementation and Obesity-Related Indices

**Figure 2:**
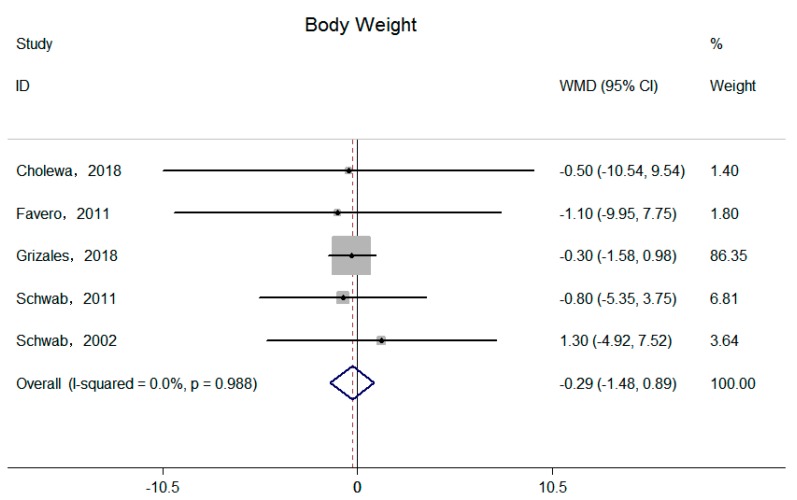
Effects of supplemental betaine on body weight in participants. The pooled effect was calculated by using a random-effects model. The diamonds denote summary risk estimate, and horizontal lines represent 95% CI.

**Figure 3:**
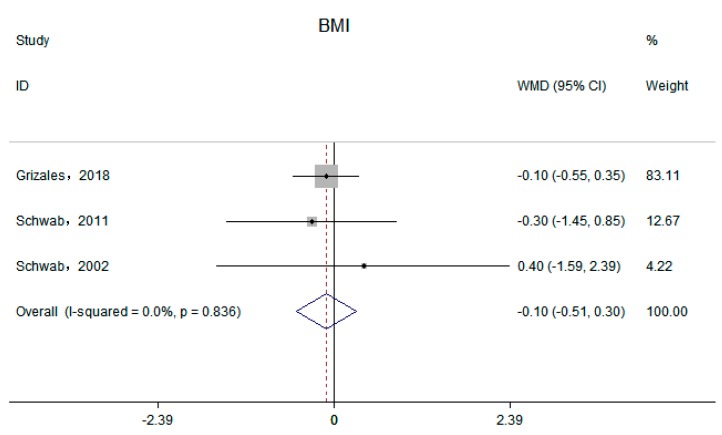
Effects of supplemental betaine on BMI in participants. The pooled effect was calculated by using a random-effects model. The diamonds denote the summary risk estimate, and the horizontal lines represent the 95% CI.

Five independent trials reported the effect of supplemental betaine on body weight [15,16,17,20,21]. The pool effect showed betaine supplementation led to no change in body weight (−0.29 kg; 95% CI: −1.48, 0.89 kg; I^2^ = 0.00%, P _for heterogeneity_ = 0.99) (Figure 2). Three trials reported the effect of supplemental betaine on BMI [15,17,21], and the pooled effect was non-significant (−0.10 kg/m^2^; 95% CI: −5.1, 0.31 kg/m^2^; I^2^ = 0.00%, P _for heterogeneity_ = 0.84) (Figure 3). Two trials reported the effect of supplemental betaine on waist circumference [15,21], and no change was observed on the pooled effect (0.68 cm; 95% CI: −1.72, 3.09 cm; I^2^ = 0.00%, P _for heterogeneity_ = 0.83). 

**Figure 4:**
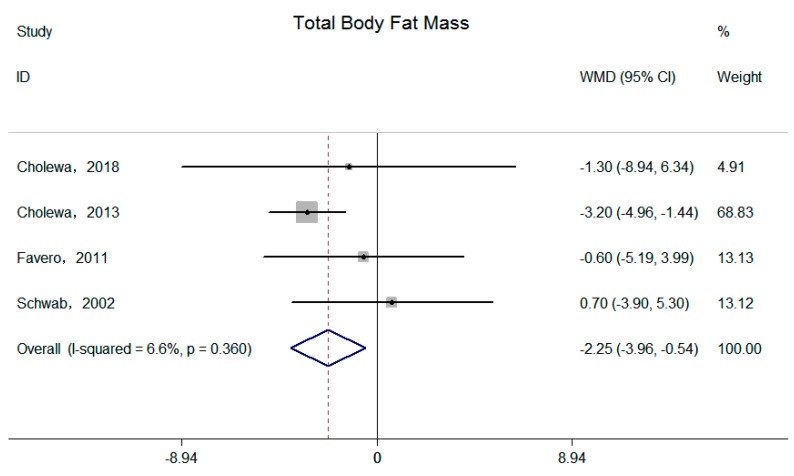
Effects of supplemental betaine on total body fat mass in participants. The pooled effect was calculated by using a random-effects model. The diamonds denote the summary risk estimate, and the horizontal lines represent the 95% CI.

**Figure 5:**
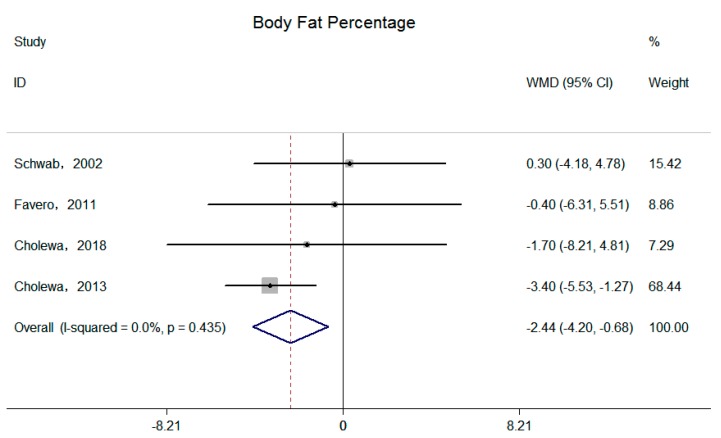
Effects of supplemental betaine on body fat percentage in participants. The pooled effect was calculated by using a random-effects model. The diamonds denote the summary risk estimate, and the horizontal lines represent the 95% CI.

Four independent trials reported supplemental betaine on total body fat mass and body fat percentage [15,16,18,20]. Significant reductions of the pooled effects were observed on both total body fat mass (−2.53 kg; 95% CI: −3.93, −0.54 kg; I^2^ = 6.6%, P _for heterogeneity_ = 0.36) (Figure 4) and body fat percentage (−2.44%; 95% CI: −4.20, −0.68%; I^2^ = 0.0%, P _for heterogeneity_ = 0.44) (Figure 5).

### 3.4. Publication Bias

Results from Begg’s rank correlation test indicated that no obvious publication bias was detected in the meta-analysis of body weight (P = 1.000), BMI (P =1.000), total body fat mass (P = 0.734) and total body fat percentage (P = 1.000) (Table 2). 

## 4. Discussion

The effects of betaine on obesity and body composition have been discussed for decades and inconsistent results have been acquired from limited human studies [19]. To our knowledge, this is the first meta-analysis of RCT studies regarding the effect of betaine on obesity in humans. The results showed that supplemental betaine significantly reduced the total body fat mass and percentage in humans, but it had no significant effects on body weight and BMI.

Obesity is a status of excess body fat accumulated in the body. Numerous indices have been employed to evaluate obesity, including body weight, BMI, waist circumference and body composition, etc. However, body weight, BMI, and waist circumference could not distinguish body lean or obese mass, leading to the limitation of accurately accessing obesity based on these criteria. Body composition, which provides the data of body fat mass and percentage, is recognized as the most accurate index to access obesity [28,29]. Betaine is a commonly used additive in animal’s feed and has been proven to decrease fat mass and increase lean mass in pigs [30,31] and chickens [13,32]. Studies in rodents also indicated a significant effect of betaine on improving body composition [11]. Consistent with these results from animals, the current review supports the findings that betaine might be used as potential dietary supplementation to improve body composition in humans. In the present meta-analysis, significant reductions in body fat mass and percentage were observed, indicating an improved effect of betaine on body composition in humans. Cholewa et al. also demonstrated that betaine supplementation significantly increased lean mass in a clinical trial [18]. Decreasing body fat and increasing lean mass may explain the unchanged body weight and BMI after betaine intervention. It should be noticed that only two studies reported significant reductions in body fat, in the existing RCTs, and they were both published by Cholewa et al. [18,20]. One of the studies had a relatively high weight in the meta-analysis, which might influence the trend of the pooled effects of betaine treatment on body fat, though no publication bias was observed [18]. In addition, the two studies were performed on non-obese young adults going through periodized exercise training programs during betaine intervention. Other RCTs without exercise training did not find significant changes in obesity after betaine treatment [15,16,17,21]. Cholewa et al. also suggested that the significant reduction in body fat in their study might be attributed to the inclusion of exercise in their studies [19]. Moreover, among the six RCTs, only two trials were performed in overweight or obese subjects [15,21] and the study by Grizales et al. did not measure the body composition of the participants [21]. To reveal the true effect of betaine on excess body fat in humans, more clinical trials should be performed in overweight or obese subjects. 

Observational studies support our findings. To date, several cross-sectional studies have investigated the relationships between circulating betaine levels and obesity. A cross-sectional study in Norway showed that plasma betaine levels ware inversely correlated with BMI, waist circumference and body fat percentage in middle age and elderly subjects [33]. Chen et al. found that higher serum levels of betaine were associated with lower body fat and better profiles of body fat distribution in Chinese adults aged 40–75 years [34]. Subsequently, they also found higher serum concentrations of betaine were associated with higher lean mass percentage in the same cohort, particular in males [35]. Our previous study also showed that higher serum betaine levels are associated with better body composition in general adults of Newfoundland [36]. These findings are largely consistent, suggesting that a higher circulating betaine is associated with lower body fat. Serum betaine levels range from 20 to 70 mmol/L [37] in healthy subjects, which are largely affected by dietary food sources of betaine or direct betaine supplements. Levels of blood betaine increase in a dose-dependent manner, and reach a new steady state in a few days following changes in dietary intake of betaine-rich meals [38]. Dietary betaine supplementation also dramatically increased fasting circulating betaine levels [15,17]. Thus, higher serum betaine levels in subjects with better profiles of body composition may be due to the higher dietary or supplementary betaine intake. To date, only one study has investigated the associations between dietary betaine intake levels and obesity-related indices, which suggested a significant negative relationship [39]. 

**Figure 6:**
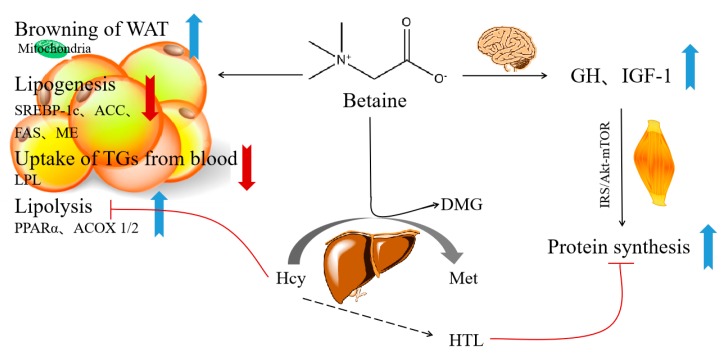
Potential mechanisms of how dietary betaine prevents obesity. PPARα, peroxisome proliferator-activated receptor α; ACOX 1/2, acyl-CoA oxidase 1/2; SREBP-1c, element-binding protein-1c; ACC, acetyl-CoA carboxylase; FAS, fatty acid synthase; ME, malic enzyme; LPL, lipoprotein lipase; WAT, white adipose tissue; Hcy, homocysteine; DMG, dimethylglycine; Met, methionine; GH, growth hormone; IGF-1, insulin-like growth factor 1; HTL, homocysteine thiolactone.

Considering that the precise mechanisms of dietary betaine protecting against obesity have not been elucidated yet, the possible mechanisms are summarized from in vitro and animal studies as follows (Figure 6). (1) Betaine supplementation could improve lipid lipolysis and reduce lipogenesis. Betaine intervention up-regulated expression of peroxisome proliferator-activated receptor α (PPARα) and downstream fatty acid oxidation-related genes, such as acyl-CoA oxidase 1/2 (ACOX 1/2) [11]. Studies also indicated that betaine increased fatty acid β-oxidation by enhancing muscle carnitine accretion, thus increasing carnitine palmitoyl transferase I-mediated free fatty acid translocation into mitochondria [12,40]. Betaine can also reduce the capacity for fatty acid and triglyceride synthesis by decreasing the activity of sterol regulatory element-binding protein-1c (SREBP-1c), acetyl-CoA carboxylase (ACC), malic enzyme (ME) and fatty acid synthase (FAS) in adipose tissue [12,32] and muscle [11]. (2) Betaine decreases uptake of triglycerides from circulating lipoproteins in adipocytes by reducing the expression of lipoprotein lipase (LPL) [32]. (3) Betaine might increase mitochondrial content and activity in hepatocytes [41] and adipocytes [11], and promote browning of white adipose tissue (WAT) [11]. (4) Betaine supplementation could decrease the levels of homocysteine (Hcy) via transmethylation of Hcy to methionine (Met), which was reported to suppress lipolysis in adipocytes [42]. (5) Betaine promotes protein synthesis. Betaine supplementation stimulates growth hormone (GH) and insulin-like growth factor 1 (IGF-1) secretion and subsequently improves the insulin IRS/Akt-mTOR protein synthetic pathway [31,43]. Betaine may also increase muscle mass by reducing homocysteine thiolactone (HTL), which is a toxic metabolite of excess homocysteine. HTL can inhibit protein synthesis via suppressing insulin/IGF-1 pathway [44].

Although our results supported the potential beneficial effect of betaine treatment on reducing body fat, possible contraindications of betaine supplementation should be considered. As a nutrient that can be acquired from either direct dietary intake or oxidation of choline, betaine is not recognized as an essential nutrient. Thus, the recommended daily intake dose and maximum daily intake dose have not been established for betaine. Recent publications indicated that dietary betaine may be metabolized by intestinal microbes to form trimethylamine (TMA), which is subsequently absorbed and metabolized by the hepatic flavin-containing monooxygenase family of enzymes (FMOs) into trimethylamine N-oxide (TMAO) [45]. TMAO is identified as a novel risk factor for cardiovascular disease (CVD) [45]. In addition, the literature reported that dietary betaine could decrease serum homocysteine levels, which is also a risk factor for CVD [10]. To date, some evidence from observational studies suggested that dietary betaine intakes were associated with increased CVD risk in humans [46]. However, most of the findings indicated that higher betaine intakes were associated with lower risk of CVD [4,47,48]. Further interventional studies are needed to verify the safety of betaine supplementation.

Several limitations of this study should be acknowledged. Firstly, the dose of betaine supplementation ranged from 2 to 9.9 g per day, the age of the participants ranged from 21 to 58.9 and the duration of intervention ranged from 10 days to 24 weeks. Due to the limited trials, it was difficult to perform subgroup studies and meta-regression. In addition, visceral fat and central obesity are more related to health compared with total body fat [49]. Moreover, body fat distribution was rarely considered in the existing RCTs. Secondly, only two of the existing RCTs were performed in overweight or obese subjects. However, the others were all conducted in healthy subjects. Therefore, additional RCTs with larger sample sizes, accurate evaluation of body fat distribution and multiple obese statuses should be performed to clarify these associations.

## 5. Conclusions

In conclusion, this systematic review and meta-analysis provided further evidence that supplemental betaine could reduce body fat in human subjects. Dietary betaine supplementation might be an effective approach for treatment of obesity and improving body composition. Given the favorable benefits identified through the meta-analysis, additional RCTs with larger sample sizes, comparable doses, wider range of ages and various obese statuses are warranted to make further insights into this topic.

## Figures and Tables

**Table 1:** Characteristics of the six eligible RCTs with betaine supplementation.

Author, Year	Country	No. (Control/Intervention)	Gender (F/M); Mean Age	Training	Duration	Study Design	Dose of Betaine Intake	Jadad Score
Cholewa et al., 2018 [20]	USA	23 (12/11)	(23/0) 21.0 ± 1.4	Yes	9 weeks	Parallel	2.5 g/day	4
Cholewa et al., 2013 [18]	USA	23(12/11)	18~35	Yes	6 weeks	Parallel	2.5 g/day	3
Favero et al., 2011 [16]	Brazil	17 (8/9)	18~30	None	10 days	Parallel	2.0 g/day	4
Grizales et al., 2018 [21]	USA	27 (13/14)	(8/19) 58.9 ± 7.67	None	12 weeks	Parallel	9.9 g/day	4
Schwab et al., 2011 [17]	Finland	63 (31/32)	(50/13) 27.0 ± 8.0	None	24 weeks	Parallel	4 g/day	4
Schwab et al., 2002 [15]	Finland	42 (20/22)	(28/14) 44.2 ± 8.7	None	12 weeks	Parallel	6 g/day	4

Abbreviations: F, female; M, male; No., number of included subjects.

**Table 2:** Results of publication bias test.

	No of Trials	Pooled Effect (95% CI)	P for Begg’s Test
Body Weight (kg)	5	−0.293 (−1.480, 0.894)	1.000
BMI (kg/m^2^)	3	−0.104 (−0.513, 0.305)	1.000
Body fat mass (kg)	4	−2.253 (−3.963, −0.544)	0.734
Body fat percentage (%)	4	−2.44 (−4.198, −0.682)	1.000
